# Visual impairment in mucopolysaccharidosis VI

**DOI:** 10.1002/jmd2.12351

**Published:** 2023-01-19

**Authors:** Augusto Monteiro Magalhães, Ana Filipa Moleiro, Esmeralda Rodrigues, Sérgio Castro, José Fonseca, Elisa Leão‐Teles

**Affiliations:** ^1^ Department of Ophthalmology São João University Hospital Center Porto Portugal; ^2^ Reference Centre of Inherited Metabolic Diseases, Pediatric Department São João University Hospital Center Porto Portugal; ^3^ Department of Surgery and Physiology Faculty of Medicine of University of Porto Porto Portugal; ^4^ Service of Pediatrics São João University Hospital Center Porto Portugal; ^5^ Department of Neuroradiology São João University Hospital Center Porto Portugal

**Keywords:** blindness, glaucoma, mucopolysaccharidosis VI, ocular involvement, optic neuropathy, posterior glaucoma

## Abstract

Mucopolysaccharidosis (MPS) VI is a rare genetic disease characterized by deficient activity of N‐acetylgalactosamine 4‐sulfatase, leading to the systemic deposition of glycosaminoglycans. Ocular involvement is classically characterized by progressive corneal clouding, ocular hypertension (OHT), and optic neuropathy. Although corneal clouding can be solved with penetrating keratoplasty (PK), visual impairment usually remains, being frequently attributed to glaucoma. The purpose of this study was to retrospectively describe a series of MPS VI patients with optic neuropathy in order to deepen the knowledge regarding the causes of severe visual impairment among these patients. We present five genetically confirmed clinical cases of MPS VI, treated with enzymatic replacement therapy, and with regular systemic and ophthalmologic follow‐up. Corneal clouding was a common early presenting feature, leading to PK in four patients. During their follow‐up, all patients developed very low visual acuities regardless of corneal grafts outcomes and controlled intraocular pressure (IOP). Furthermore, all patients exhibited optic atrophy and imagiological evidence of significant subarachnoid space enlargement and consequent optic nerve thickness reduction, suggesting compression of the optic nerve in a retro‐ocular location as the cause of optic neuropathy. Although optic neuropathy in MPS VI is commonly attributed to glaucoma due to OHT, by describing a series of five MPS VI patients, we provided evidence that, differently from glaucoma, compression of optic nerve in a retro‐ocular location is crucial for the development of optic neuropathy, at least in some cases. We propose the denomination of posterior glaucoma and suggest it as an important cause of optic neuropathy, leading to severe visual impairment and blindness among these patients.

**Synopsis**

By describing a series of five mucopolysaccharidosis VI patients, we provide evidence that, differently from glaucoma, compression of optic nerve in a retro‐ocular location is crucial for the development of optic neuropathy, at least in some cases, resulting in severe visual impairment and blindness, thus proposing the denomination of posterior glaucoma.

## 1 INTRODUCTION

Mucopolysaccharidosis (MPS) are rare genetic disorders characterized by lysosomal enzyme defects, promoting the intra and extracellular deposition of systemic glycosaminoglycans (GAGs).^1^ Depending on the affected enzyme, different types of MPS with different phenotypes may be present.^2^ Common manifestations include growth restriction, skeletal abnormalities, structural cardiac valvular disease, hearing loss, upper airway obstruction, and visual impairment resulting from different causes.^2^ Furthermore, ocular findings are often early presenting features, constituting important clues for its diagnosis.^1^, ^2^, ^3^, ^4^, ^5^, ^6^

Given the low prevalence and high mortality of MPS during childhood, research on this group of diseases have been focused mainly on lethal manifestations.^7^ However, the introduction of novel treatments led to an increase in life span and to an improvement in the quality of life of these patients.^7^

MPS type VI, also known as Maroteaux‐Lamy syndrome, is a rare autosomal recessive disease. It is characterized by deficient activity of the enzyme N‐acetylgalactosamine 4‐sulfatase resulting from mutations in the arylsulphatase B (*ARSB*) gene, leading to the deposition of the GAG dermatan‐sulfate and chondroitin‐sulfate.^8^ Patients generally present early in life with a distinctive phenotype including facial dysmorphias and respiratory disease. Other features as small stature, cardiac valve disease, sleep apnea, skeletal degeneration, medullar and peripheral compression, as well as senses impairment may develop throughout growth.^2^, ^9^ Differently from other types of MPS, MPS VI patients usually have no cognitive impairment.^2^, ^9^ Enzyme replacement therapy (ERT) is available and, if early implemented, may change the course of the disease.^8^, ^10^

The most common ocular manifestation of MPS VI is corneal clouding due to the deposition of GAG, which frequently leads to corneal penetrating keratoplasty (PK).^2^, ^3^ However, although PK allows early recovery of vision to a certain extent, most patients end up developing low visual acuities frequently attributed to ocular hypertension (OHT) and optic neuropathy.^1^, ^4^, ^5^, ^8^

The cause of optic neuropathy is probably multifactorial. Glaucoma and OHT are commonly present in these patients. OHT may be due to the infiltration of trabecular meshwork with consequent aqueous humor' low drainage.^2^, ^5^ However, OHT may also be an artefactual measurement due to the high corneal thickness caused by GAG deposition.^6^ On the other hand, orbital and retro‐orbital factors may also contribute to optic neuropathy and atrophy. Indeed, the increase in intra‐cranial pressure and the posterior compression of optic nerve may be critical in the progression of optic neuropathy.

In this work, we retrospectively described a series of five MPS VI patients with optic neuropathy, aiming to deepen the knowledge regarding the causes of severe visual impairment and blindness among these patients. Differently from glaucoma, our observations suggest that compression of optic nerve in a retro‐ocular location (i.e., posterior to the globe) is crucial for the development of severe optic neuropathy. We therefore proposed the denomination of posterior glaucoma as an important cause of optic neuropathy, leading to visual impairment and blindness in MPS VI patients.

## 2 PATIENT INFORMATION

Five patients were identified with a diagnosis of MPS VI and optic neuropathy, in outpatient follow‐up at the Inherited Metabolic Diseases Center of São João University Hospital Center, Porto, Portugal. All patients were treated with ERT, and the follow‐up ranged from 13 to 19 years. Also, all patients had a distinctive biochemical profile, with positive urinary GAGs, and enzyme and genetic diagnosis of MPS VI.

Table 1 presents the summary of the general information of each MPS VI patient.

**TABLE 1:** Summary of the general information of each MPS VI patient

Case	Sex	Age at diagnosis	Lifespan	*ARSB* mutation			Phenotype
				Nucleotide change	Amino acid change	Genotype	
Patient 1	F	16 mo	28 y^a^	c.944G>A	p.R315Q	Homozygosis	Severe
Patient 2	M	10 mo	28y^a^	c.160G>A	p.D54N	Homozygosis	Severe
Patient 3	F	28 mo	32 y 10 mo^b^	c.215T>G	p.L72R	Homozygosis	Severe
Patient 4	F	16 y	29 y^b^	c.944G>A	p.R315Q	Homozygosis	Severe
Patient 5	M	11 y	26 y 1 mo^b^	c.944G>A	p.R315Q	Homozygosis	Severe

Abbreviations: F, female; M, male; mo, months; y, years.

^a^ Living patients.

^b^ Deceased patients.

Three patients were female and two were male, with an age at diagnosis ranging from 10 months to 16 years. Three patients carried the mutation c.944G>A (p.R315Q) and all patients presented severe phenotypes (Table 1).

All patients started ERT with galsulfase 1 mg/kg/week as soon as it was available in our center (four patients) or immediately after diagnosis (one patient) (mean age at the start of ERT: 11 years and 1 month, range 2–18 years). ERT was given continuously to all patients with a duration ranging from 11 years and 1 month to18 years and 4 months.

## 3 CLINICAL FINDINGS, DIAGNOSTIC ASSESSMENT, FOLLOW‐UP, AND OUTCOMES

### 3.1 Multisystemic involvement

Our patients were characterized by the typical severe and progressive multisystemic involvement. Although all patients were considered normal during neonatal period, at least two patients showed some distinctive facial features in photographs at these stages. Also, all patients had recurrent ear, nose, and throat infections during the first year of life.

The five patients presented the characteristic phenotype consisting of coarse features, short stature, skeletal involvement with dysostosis multiplex, stiffness and joint contractures, claw hands, organomegaly, hernias, and reduced mobility. Progressive mixed hearing loss and involvement of the other senses, namely visual impairment, were also described in all patients as well as speech difficulties due to vocal cords involvement with deposition of GAGs.

The progressive deterioration of cardiorespiratory function is a key feature of MPS VI.^8^, ^10^ Cardiac involvement was present in all patients with different degrees of severity, being mitral and aortic thickening/dysplasia the most relevant findings.

A mixed restrictive/obstructive respiratory pattern was described in all patients who presented severe sleep apnea syndrome needing nocturnal noninvasive ventilator support. Tracheostomy was performed in two patients due to significant respiratory obstruction: one patient before ERT availability, at 8 years old, and the other patient at the age of 15, 3 months after ERT initiation.

Considering neurologic commitment, all patients exhibited cervical medullar compression and four patients showed peripheral nerve compression. Furthermore, epilepsy was described in two patients. Although only two patients had the diagnosis of marked hydrocephalus, all presented imagiological evidence of subarachnoid space enlargement.

According to previous reports, important cognitive impairment was not found in any patient, although three patients had suboptimal school performance related to the severe phenotype of the disease with multisystemic involvement.

All patients were submitted to multiple surgical interventions for ear, nose, and throat involvements, hernias, and for the management of orthopedic or neurological problems. Furthermore, all patients developed progressive limitations in their activities of daily living during their life course, leading to the need of different technical devices.

Although ERT allows an increase in life expectancy, three patients died in the third and fourth decades of live: one due to cardiorespiratory insufficiency and two patients with pneumonia.

### 3.2 Ophthalmologic involvement

Table 2 shows the summary of the ophthalmologic features and therapeutic interventions for each MPS VI patient.

**TABLE 2:** Summary of the ophthalmologic features and therapeutic interventions for each MPS VI patient

Case	Cornea commitment			IOP		Optic nerve commitment
	Opacification	PK	Post‐PK	Baseline	Post‐PK	
Patient 1	Yes	Yes	Transparent graft	OHT, with mean values of 25–27 mmHg with a combination of dorzolamide hydrochloride 2.0% and timolol maleate 0.5%	Mean values of 12–13 mmHg without treatment	Optic neuropathy
Patient 2	Yes	No	NA	OHT, with maximum values of 33–34 mmHg without treatment and 20 mmHg on both eyes with latanoprost 0.005% and a combination of dorzolamide hydrochloride 2.0% and timolol maleate 0.5%	NA	Optic neuropathy
Patient 3	Yes	Yes	Transparent graft	OHT, with mean values of 26–27 mmHg with a combination of dorzolamide hydrochloride 2.0% and timolol maleate 0.5%	Mean values of 13–14 mmHg without treatment	Optic neuropathy
Patient 4	Yes	Yes	Re‐opacification	Mean values of 20 mmHg on both eyes without treatment	Mean values of 15–16 mmHg with timolol maleate 0.5%	Optic neuropathy
Patient 5	Yes	Yes	Re‐opacification	OHT with mean values of 28 mmHg without treatment	Mean values of 24–25 mmHg with com an association of dorzolamide hydrochloride 2.0% with timolol maleate 0.5%	Optic neuropathy

Abbreviations: IOP, intraocular pressure; MPS, mucopolysaccharidosis; NA, not applicable; OHT, ocular hypertension; PK, penetrating keratoplasty.

All patients presented corneal clouding due to GAG accumulation as the first ocular manifestation. During ophthalmologic follow‐up, all patients exhibited progressive corneal opacity and thickening, with maximum pachymetry values of 1500 μm, a value almost three times superior comparing to the normal cornea. Due to severe corneal opacification and consequent low visual acuity, PK was performed in four of the five patients (Table 2). PK was also proposed for the fifth patient, however the patient and his family denied the procedure due to the anesthetic risks.

Before PK, the best corrected visual acuity (BCVA) ranged from hands motions to 2/10 (decimal scale). PK allowed only a slight vision recovery in all patients, with a gain of one line on average. Corneal grafts remained transparent during the entire follow‐up in two patients, with no signs of rejection or relapse of the disease. The other two patients subjected to transplantation presented with opacified grafts during follow‐up.

OHT was another remarkable finding in these patients, who developed this feature during follow‐up with baseline values up to 34 mmHg (iCare R tonometry), leading to the need of topical medication to achieve intraocular pressure (IOP) control.

After PK, two patients maintained controlled IOP, with values below 20 mmHg without hypotensive medication. The two patients who had relapse of opacification of the cornea also developed OHT after PK. However, one patient achieved IOP values below 20 mmHg with timolol maleate 0.5%, and the other patient with a combination of dorzolamide hydrochloride 2.0% and timolol maleate 0.5%.

The patient who denied PK maintained controlled IOP values of 20 mmHg (iCareR tonometry) on both eyes with topical medication (latanoprost 0.005% and a combination of dorzolamide hydrochloride 2.0% and timolol maleate 0.5%) (Table 2).

Despite IOP control and PK, all patients presented severe visual impairment during follow‐up, with BCVA of only light perception or hand motion at the end of follow‐up.

### 3.3 Optic nerve involvement and imageology

**FIGURE 1:**
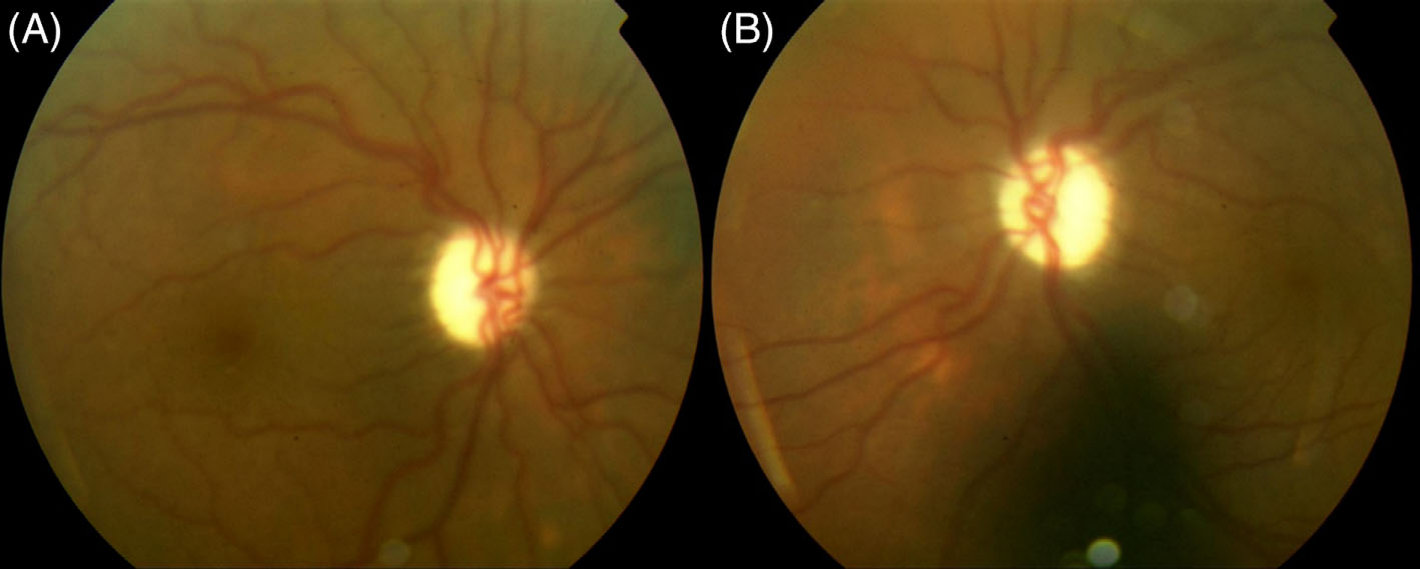
Optic nerve retinography of (A) right and (B) left eyes of patient number 1, highlighting optic disk pallor with no significant cupping

Figure 1 shows optic nerve retinography of both eyes of patient 1, highlighting optic disk pallor with no significant cupping. In fact, progressive optic nerve pallor was evident in all patients, without observed hemorrhages or papilledema during follow‐up.

**FIGURE 2:**
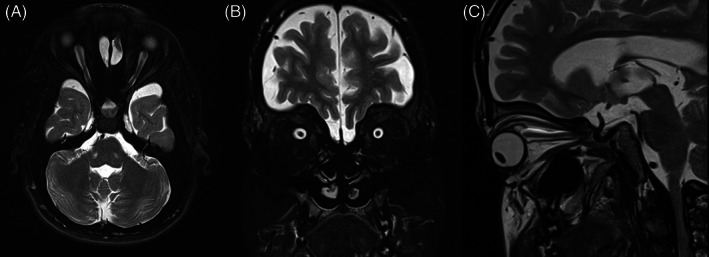
T2‐fat saturation MRI for patient 1 showing enlarged perioptic subarachnoid space. (A) Axial section; (B) coronal section; (C) sagittal section

**FIGURE 3:**
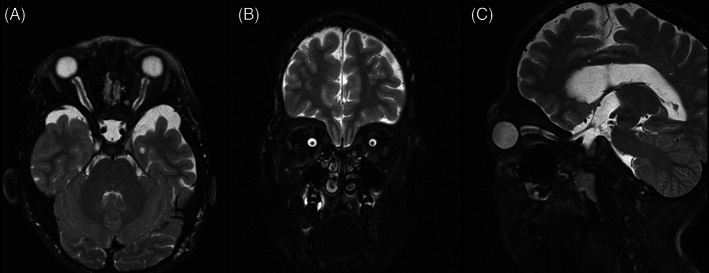
T2‐fat saturation MRI for patient 2 showing enlarged subarachnoid space with the “sausage” sign in sagittal section. (A) Axial section; (B) coronal section; (C) sagittal section

**FIGURE 4:**
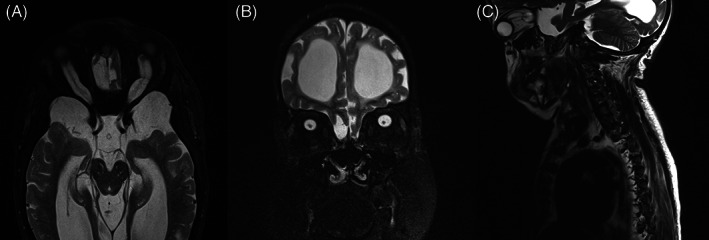
T2‐fat saturation MRI for patient 3 showing exuberant enlargement of subarachnoid space with “sausage” sign in axial section and reduced thickness of optic nerves, more pronounced on the left. (A) Axial section; (B) coronal section; (C) sagittal section

**FIGURE 5:**
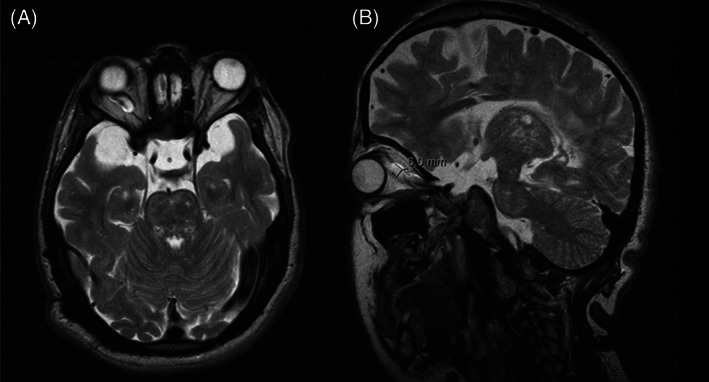
T2 ponderation MRI for patient 4 with evidence of enlargement of perioptic subarachnoid space. (A) Axial section. (B) Sagittal section

**FIGURE 6:**
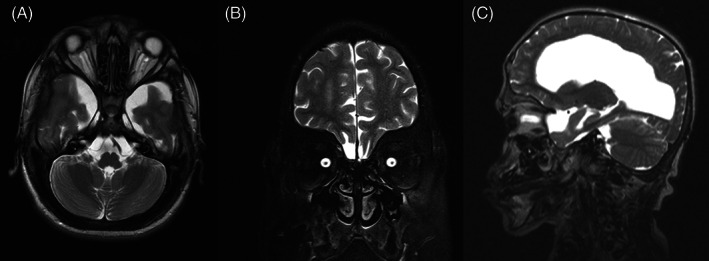
T2‐fat saturation MRI for patient 5 showing enlarged perioptic subarachnoid space with reduced optic nerve thickness. (A) Axial section; (B) coronal section; (C) sagittal section

Figures 2, 3, 4, 5, and 6 present orbital magnetic resonance imaging (MRI) results for each MPS VI patient.

MRI revealed a clear enlargement of the subarachnoid space around optic nerves and a reduction in optic nerves thickness in all patients. The persistent enlargement of this space and the consequent reduction in optic nerve thickness confirm that optic atrophy is a hallmark of these patients and suggest retro‐orbital pressure as the main cause of this neuropathy.

MRI also showed significant volume reduction of the optic chiasm, band, and optic nerves accompanied by an enlargement of the surrounding cisterns and increased amount of cerebrospinal fluid inside the sheaths (Figures 2, 3, 4, 5, and 6), suggesting optic atrophy in an environment of prominent perivascular spaces with compression of the surrounding parenchyma and interstitial cerebral edema.

## 4 DISCUSSION

In this work, we retrospectively described a series of five MPS VI patients with optic neuropathy, aiming to deepen the knowledge regarding the causes of severe visual impairment and blindness among these patients.

MPS, and particularly MPS VI, are a group of disorders in which ocular involvement is pronounced and also an early presenting feature.^1^, ^2^, ^3^, ^4^, ^5^ Specifically regarding MPV VI, patients frequently present corneal clouding, OHT, and optic nerve anomalies.^1^, ^4^, ^5^

Besides the early and frequent ocular manifestations abovementioned, due to the increase in life span among these patients and to the emergence of new diagnostic tools, other manifestations occurring later throughout the course of MPS were identified. Our group has recently described, for the first time, the presence of scleral thickening presumably due to scleral GAG deposits in MPS VI patients.^11^ Similarly, spectral‐domain optical coherence tomography (SD‐OCT) images allowed us to show the presence of increased thickness of the external limiting membrane in the foveal area with concomitant loss of the interdigitation, ellipsoid, and myoid zones in parafoveal and perifoveal regions in MPS I^12^ and MPS II patients.^13^ Furthermore, we also reported the increase of choroidal thickness probably related to GAG deposition in a MPS IV patient.^14^

Therefore, new perspectives on these diseases emerge. In MPS VI, corneal clouding has been the most common ocular finding described, and frequently regarded as the leading cause of blindness.^15^ Corneal clouding can be solved with PK improving the vision, at least immediately after surgery, in most patients.^16^ However, MPS VI patients frequently experience very low visual acuities after successful PK.^16^ In fact, in our series of patients, regardless of graft results after PK, all patients presented very low visual outcomes, suggesting that different etiologies other than cornea opacity contribute to low visual acuity.

Optic neuropathy is also an important cause of low visual acuities,^1^, ^3^, ^5^ which has been classically attributed to glaucoma.^7^ The prevalence of glaucoma ranges from 2.1 to 12.5% in all MPS, being considered relatively common in MPS VI. The progressive neuropathy is presumably attributed to OHT, which is also a common finding in MPS VI patients.^7^ In these patients, OHT may result from two mechanisms: deposition of GAGs in the trabecular meshwork decreasing the outflow of aqueous humor; or deposition of GAGs in the cornea and iris with narrowing of the camerular angle and consequent angle‐closure glaucoma.^1^, ^2^, ^15^ However, GAG deposition in the eye may also affect the tensile and viscoelastic behavior of the sclera, as well as the cornea.^15^ Therefore, it is important to take into account that high IOP may also be an artifact attributed to thickened and stiffened corneas.

The importance of glaucoma and OHT in the development of severe visual impairment and blindness may thus be questionable. Firstly, as already mentioned, OHT may constitute an artifact attributed to thickened and stiffened corneas, not reflecting a real pressure.^6^ Secondly, the real impact of potential OHTs may also be subject of debate.

Indeed, although optic neuropathy is frequently attributed to glaucoma, other causes including compression of optic nerve in a retro‐ocular location may also contribute to optic neuropathy. The deposition of GAGs in the nerve may occur promoting its atrophy, and the thickening of dura sheaths may promote nerve compression. Furthermore, MPS VI patients frequently experience chronic hydrocephaly which may also contribute to posterior compression of the nerve.^1^, ^4^, ^5^

We presented five cases of MPS VI patients in which the hypothesis of glaucoma as the main cause of optic neuropathy due to OHT becomes unlikely, given that: (1) imagological signs of optic atrophy were evident in all MPS VI patients, although without fundoscopic papilledema or hemorrhages; (2) the atrophic optic nerves did not present the typical glaucoma cupping; (3) all patients maintained controlled IOP during follow‐up; and (4) all patients exhibited evidence of subarachnoid space enlargement and consequent optic atrophy on MRI.

Therefore, our observations suggest that, differently from glaucoma, in which the compression on nerve fibers occurs within the globe, compression of the optic nerve in a retro‐ocular location may be a cause of optic neuropathy as important as, or more important than glaucoma, resulting in severe visual impairment and blindness. We believe that the chronic hydrocephaly experienced during these patients' lives caused a constant posterior pressure on the optic nerve that led to progressive atrophy without the development of papilledema in some cases. We thus suggest this mechanism as an important cause of severe visual impairment and blindness, proposing the designation of posterior glaucoma, given the location of the compression of the fibers that form the optic nerve. This manifestation is often diagnosed late in the course of the disease and may also be considered as a late ocular manifestation of MPS VI. Therefore, in cases of very low acuity or sudden vision loss, urgent neuroimaging should be performed in these patients to clarify the presence of severe optic nerve damage by retroocular mechanisms.

The main strengths of this work were the inclusion of five clinical cases of MPS VI, which is a high number of patients considering that it is a rare disease, and the inclusion of a neuroradiological study for each patient. On the other hand, this work had some limitations, including the retrospective observation of patients and the limited number of patients included, nevertheless high for a rare disease.

## 5 CONCLUSION

We described a series of five MPS VI patients highlighting the relevance of retro‐ocular compression of the optic nerve on visual impairment in these patients. To the best of our knowledge, this was the first case series consistently showing this finding and suggesting a new designation—posterior glaucoma—as a crucial cause of severe visual impairment and blindness in MPS VI patients.

## AUTHOR **CONTRIBUTIONS**

Augusto Monteiro Magalhães and Ana Filipa Moleiro were involved in study design, data collection, analysis, and interpretation, and in the drafting of the manuscript. Esmeralda Rodrigues, Sérgio Castro, José Fonseca, and Elisa Leão‐Teles were involved in data collection, analysis, and interpretation. All authors were involved in the critical revision of the manuscript. All authors approved the final version of the manuscript and take responsibility for the accuracy or integrity of any part of the work.

## FUNDING INFORMATION

This work was supported by BioMarin by funding medical writing assistance provided by Scientific ToolBox Consulting (Lisbon, Portugal). The authors confirm independence from the sponsors; the content of the article has not been influenced by the sponsors.

## CONFLICT OF INTEREST

Augusto Monteiro Magalhães received a speaker honorarium from Biomarin. Elisa Leão‐Teles received support to attend scientific meetings, consulting fees, or speaker honoraria from Sanofi Genzyme, Biomarin, and Takeda. Ana Filipa Moleiro, Esmeralda Rodrigues, Sérgio Castro, and José Fonseca declare that they have no conflict of interest.

## ETHICS APPROVAL

Ethics approval was not required for this work.

## INFORMED CONSENT

All procedures followed were in accordance with the ethical standards of the responsible committee on human experimentation (institutional and national) and with the Helsinki Declaration of 1975, as revised in 2000. Informed consent was obtained from all patients for being included in the study.

## ANIMAL RIGHTS

This article does not contain any studies with animal subjects performed by the any of the authors.

## Data Availability

All data relevant to the study are included in the article. Further information can be obtained from the corresponding author.
